# Electronic Cigarette Exposure Increases the Severity of Influenza a Virus Infection via TRAIL Dysregulation in Human Precision-Cut Lung Slices

**DOI:** 10.3390/ijms24054295

**Published:** 2023-02-21

**Authors:** Hina Agraval, Taylor Crue, Niccolette Schaunaman, Mari Numata, Brian J. Day, Hong Wei Chu

**Affiliations:** 1 Department of Medicine, National Jewish Health, 1400 Jackson Street, Denver, CO 80206, USA; 2 School of Medicine, University of Colorado, 12700 E 19th Ave, Aurora, CO 80045, USA

**Keywords:** electronic cigarettes, PCLS, Influenza A virus, TRAIL

## Abstract

The use of electronic nicotine dispensing systems (ENDS), also known as electronic cigarettes (ECs), is common among adolescents and young adults with limited knowledge about the detrimental effects on lung health such as respiratory viral infections and underlying mechanisms. Tumor necrosis factor (TNF)-related apoptosis-inducing ligand (TRAIL), a protein of the TNF family involved in cell apoptosis, is upregulated in COPD patients and during influenza A virus (IAV) infections, but its role in viral infection during EC exposures remains unclear. This study was aimed to investigate the effect of ECs on viral infection and TRAIL release in a human lung precision-cut lung slices (PCLS) model, and the role of TRAIL in regulating IAV infection. PCLS prepared from lungs of nonsmoker healthy human donors were exposed to EC juice (E-juice) and IAV for up to 3 days during which viral load, TRAIL, lactate dehydrogenase (LDH), and TNF-α in the tissue and supernatants were determined. TRAIL neutralizing antibody and recombinant TRAIL were utilized to determine the contribution of TRAIL to viral infection during EC exposures. E-juice increased viral load, TRAIL, TNF-α release and cytotoxicity in IAV-infected PCLS. TRAIL neutralizing antibody increased tissue viral load but reduced viral release into supernatants. Conversely, recombinant TRAIL decreased tissue viral load but increased viral release into supernatants. Further, recombinant TRAIL enhanced the expression of interferon-β and interferon-λ induced by E-juice exposure in IAV-infected PCLS. Our results suggest that EC exposure in human distal lungs amplifies viral infection and TRAIL release, and that TRAIL may serve as a mechanism to regulate viral infection. Appropriate levels of TRAIL may be important to control IAV infection in EC users.

## 1. Introduction

Cigarette smoking is a well-recognized risk factor for various lung pathologies including chronic obstructive pulmonary disease (COPD), respiratory infections, asthma, and lung cancer [1,2]. Electronic cigarettes (ECs) are commonly used by youth and young adults with a rapidly increasing number of users. E-cigarette or vaping product use-associated lung injury (EVALI) was first reported in 2019–2020 in the USA [3]. This outbreak resulted in 68 deaths and 2807 hospitalizations and raised concern about the safety and hazardous effects of vaping on health. However, the adverse effects and underlying mechanisms of vaping on lung health, especially on distal lungs are poorly understood [4]. Recently, various in vitro studies reported detrimental effects of ECs including inflammation, cytotoxicity, and oxidative stress in different types of cells [5,6,7,8]. In a murine model study, EC exposure induced airway inflammation in both control and asthmatic groups [9]. In another study, acute exposure to ECs increased the release of pro-inflammatory cytokines IL-6 and IL-1β in mice whereas chronic EC exposure was associated with more severe effects including chronic inflammation and emphysema [10].

Influenza A virus (IAV), a respiratory pathogen from the Orthomyxoviridae family, is known to contribute to COPD exacerbations [11,12]. It has been suggested that cigarette smoke may increase the risk of respiratory viral infections [13]. However, whether EC exposures directly increase the risk of IAV infection in human distal lungs, the site of emphysema, remains unclear. Recently, a study from our group reported that EC exposure aggravates the pro-inflammatory response of human distal airway epithelium during IAV infection [8]. A limitation of our previous study was that it only examined airway epithelial cells but not the entire human lung tissue. Precision-cut lung slices (PCLS) are uniform tissue slices representing an ex vivo organotypic model [14]. PCLS retains a three-dimensional tissue structure including small airways, lung parenchyma, mechanical properties, and other organ-specific features. It also contains most types of lung cells including fibroblasts, alveolar type I (ATI) and alveolar type II (ATII) cells, monocytes, macrophages, T cells, and natural killer cells, which makes PCLS an ideal model to mimic human lung responses to EC exposures and viral infection [14,15].

The mechanisms by which ECs impact IAV infection have not been well explored. IAV infection is known to induce apoptosis in infected epithelial cells as a defense mechanism to eliminate infected/damaged cells [16]. A key apoptotic pathway is related to the activation by tumor necrosis factor (TNF) superfamily members [17,18,19,20,21]. TNF-related apoptosis-inducing ligand (TRAIL), a type II transmembrane protein belonging to the TNF family, can be secreted by various cell types to induce apoptosis. TRAIL is up-regulated in IAV-infected epithelial cells and immune cells [22]. TRAIL binds to the cell surface receptors DR4/TRAIL-R1 and DR5/TRAIL-R2 to transduce the death signals and initiate apoptosis [23]. Levels of TRAIL and its death receptors are elevated in the airways and serum of COPD patients where TRAIL is associated with regulation of inflammation, apoptosis, and remodeling [24,25,26].

In the present study, we hypothesized that EC exposures worsen IAV infection in human PCLS through dysregulation of TRAIL expression. To test this hypothesis, we examined the effect of EC juice (E-juice) on TRAIL/TNF alpha release and viral infection and tested the role of TRAIL in IAV infection in E-juice exposed PCLS.

## 2. Results

### 2.1. E-Juice Treatment Increased IAV Levels in Human PCLS

**Figure 1:**
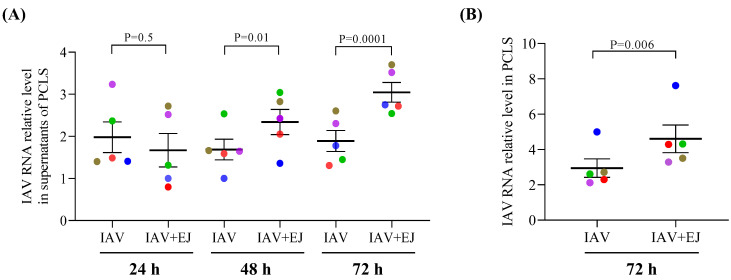
E-juice treatment increased influenza A virus (IAV) levels in human precision-cut lung slices (PCLS). PCLS were treated with IAV and E-juice (EJ) for 24, 48, and 72 h. IAV load in supernatants (**A**) and tissue samples (**B**) was analyzed by RT-PCR. Each data point (colored dot) represents the average of 3 replicates from each respective individual donor (*n* = 5 donors). Horizontal lines indicate mean ± SEM. Paired *t*-test was used to analyze the data.

Cigarette smoke exposure has been shown to increase the severity of respiratory viral infections [27]. To determine the effect of E-juice on IAV infection, we analyzed the changes in viral RNA levels in PCLS supernatants collected at 24, 48, and 72 h post-infection, and tissues collected at 72 h post-infection. In PCLS treated with IAV alone, viral load in supernatants trended to decrease over time (from 24 to 72 h). In contrast, in PCLS treated with both E-juice and IAV, viral levels in the supernatants increased from 24 h to 72 h. At 48 h, and especially at 72 h post-infection, viral load was significantly increased (~1.6 fold) in supernatants collected from PCLS exposed to both E-juice and IAV as compared to IAV infection alone. Similarly, the tissue viral level in PCLS treated with E-juice and IAV for 72 h was significantly higher (~1.5 fold) than that in PCLS treated with IAV alone (Figure 1A,B).

### 2.2. E-Juice Treatment Amplified TRAIL and TNF-α Release in IAV-Infected Human PCLS

To determine the potential mechanisms by which EC exposures increase viral levels in PCLS, we measured TRAIL release in supernatants. TRAIL-mediated immunity may be important for the host cells to clear viruses [28] as IAV titers and morbidity increased in TRAIL deficient mice as compared to wild-type mice [29].

**Figure 2:**
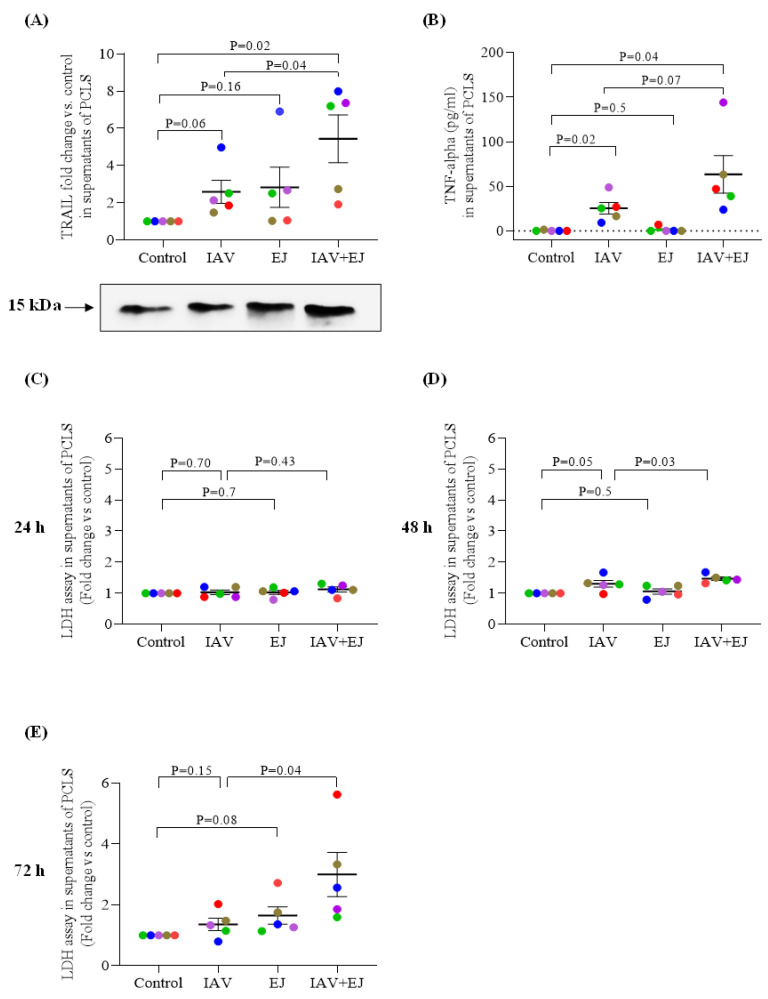
E-juice treatment increased TRAIL, TNF-α release and cytotoxicity in human precision-cut lung slices (PCLS) infected with influenza A virus (IAV). PCLS were treated with IAV and E-juice (EJ) for 24, 48, and 72 h. (**A**) TRAIL release (72 h) in supernatants analyzed by western blotting. (**B**) TNF-α release (72 h) in supernatants analyzed by ELISA. (**C**–**E**) Cytotoxicity was analyzed by performing the LDH assay. Each data point (colored dot) represents the average of 3 replicates from each respective individual donor (*n* = 5 donors). Horizontal lines indicate mean ± SEM. Paired *t*-test was used to analyze the data.

At 72 h post viral infection, E-juice alone or IAV alone moderately increased the release of TRAIL in the supernatants of PCLS. Combined E-juice and IAV treatment significantly amplified the release of TRAIL compared with the control (~5 fold) (Figure 2A).

TNF-α is mainly produced by activated macrophages, T lymphocytes, and natural killer cells during acute inflammation [30]. TNF-α is involved in the pro-inflammatory response to cigarette smoke exposure [31,32]. TNF-α may induce apoptosis of various types of cells under pathological conditions such as viral infection. To examine the effect of E-juice on TNF-α release, TNF-α in supernatants of human lung PCLS were measured after 72 h of viral infection. IAV infection alone increased TNF-α release which was amplified by E-juice (~2.5-fold increase vs. IAV treatment alone) (Figure 2B).

### 2.3. E-juice Treatment Increased Cytotoxicity in IAV-Infected Human PCLS

Having shown increased TRAIL and TNF in PCLS treated with both E-juice and IAV, we evaluated their effect on cytotoxicity. Cellular LDH release into supernatants of PCLS were used to indicate the cytotoxic effect of E-juice and IAV treatment. The level of LDH was increased by IAV alone at 48 h, which was moderately enhanced by E-juice. E-juice increased LDH levels in IAV-infected PCLS after 72 h of infection (~2.2-fold increase vs. IAV treatment alone) (Figure 2C–E). Overall, these results suggest that E-juice may enhance cell injury during IAV infection.

### 2.4. TRAIL Neutralizing Antibody Reduced IAV Release into the Supernatant but Increased Viral Load in E-Juice Exposed Human PCLS

**Figure 3:**
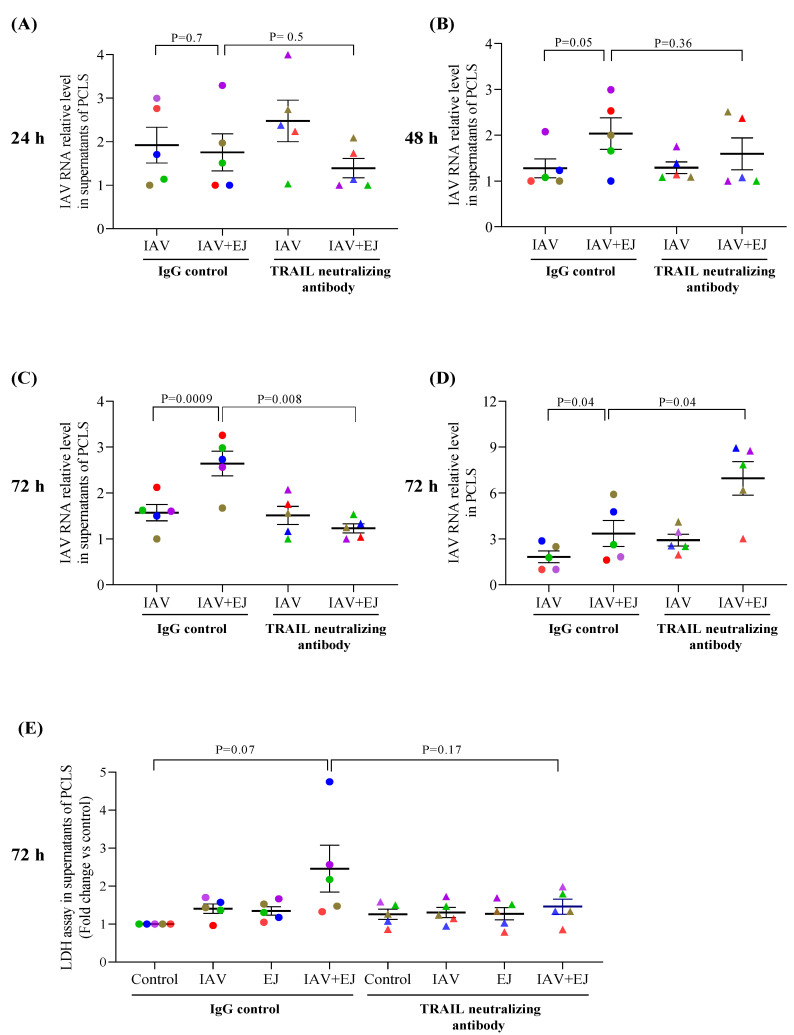
TRAIL neutralizing antibody regulated influenza A virus (IAV) infection in human precision-cut lung slices (PCLS). PCLS were treated with IAV and E-juice (EJ) in the presence or absence of a TRAIL neutralizing antibody (50 ng/mL) and IgG control (50 ng/mL) for 24, 48, and 72 h. IAV load in supernatants (**A**–**C**) and lung tissue (**D**) was analyzed by RT-PCR. Cytotoxicity (**E**) was analyzed by the LDH assay. Each data point (colored dot) represents the average of 3 replicates from each respective individual donor (*n* = 5 donors). Horizontal lines indicate mean ± SEM. Paired *t*-test was used to analyze the data.

TRAIL signaling was blocked using a TRAIL neutralizing antibody to examine the role of TRAIL in IAV release from lung tissue cells. TRAIL neutralizing antibody significantly reduced viral levels (~53% reduction over IgG control) at 72 h, but not at 24 and 48 h, in supernatants of PCLS treated with both E-juice and IAV (Figure 3A–C). In contrast, viral levels in the lung tissue were increased by the TRAIL neutralizing antibody as compared to the IgG control (~2.1 fold) (Figure 3D). The TRAIL neutralizing antibody trended to reduce the LDH levels in PCLS treated with both IAV and E-juice (Figure 3E). Our data suggest that blocking TRAIL signaling may reduce cell injury, coupled with increased intracellular viral load, while decreasing viral release into the supernatants.

### 2.5. Recombinant TRAIL Decreased Tissue Viral Load but Increased IAV Release in E-Juice-Exposed Human PCLS

**Figure 4:**
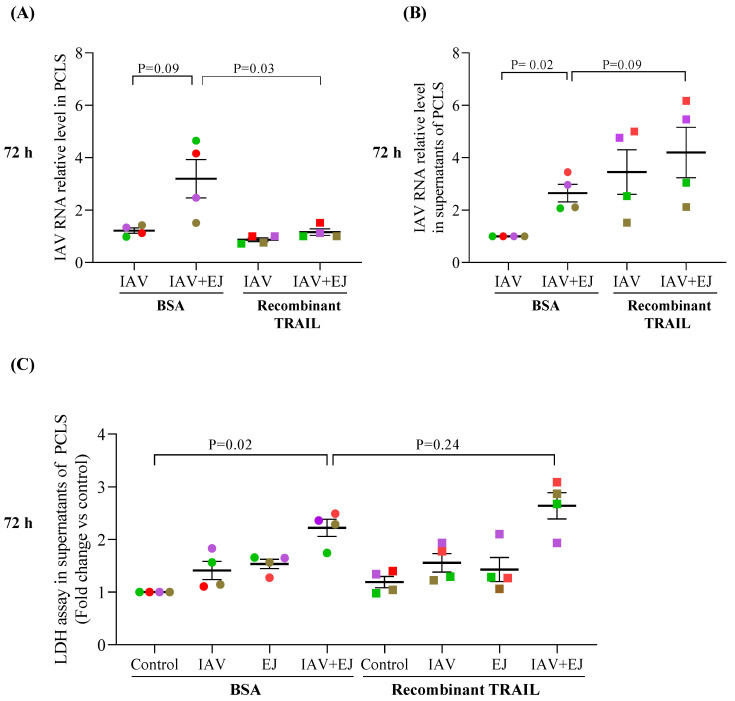
Recombinant TRAIL altered influenza A virus (IAV) infection in human precision-cut lung slices (PCLS). PCLS were treated with IAV and E-juice (EJ) in the presence or absence of BSA (control) and recombinant TRAIL (10 ng/mL) for 72 h. IAV load in lung tissue (**A**) and supernatants (**B**) was analyzed by RT-PCR. Cytotoxicity (**C**) was analyzed by the LDH assay. Each data point (colored dot) represents the average of 3 replicates from each respective individual donor (*n*  =  4 donors). Horizontal lines indicate mean ± SEM. Paired *t*-test was used to analyze the data.

To further evaluate the role of TRAIL in IAV infection, recombinant TRAIL protein (10 ng/mL) was added to PCLS treated with IAV alone or with combination of IAV and E-juice for 72 h. TRAIL, as compared to the BSA control, significantly decreased the tissue viral load in PCLS exposed to both E-juice and IAV (Figure 4A). In contrast, TRAIL increased IAV release into supernatants (Figure 4B) of PCLS treated with both E-juice and IAV (Figure 4B). TRAIL trended to increase LDH levels in PCLS treated with both E-juice and IAV (Figure 4C). Together, these data suggest that TRAIL may promote cell injury and the release of viruses from the intracellular compartment into the supernatant.

### 2.6. TRAIL Treatment Enhanced the Type I and Type III Interferon Response in PCLS Treated with E-Juice and IAV

**Figure 5:**
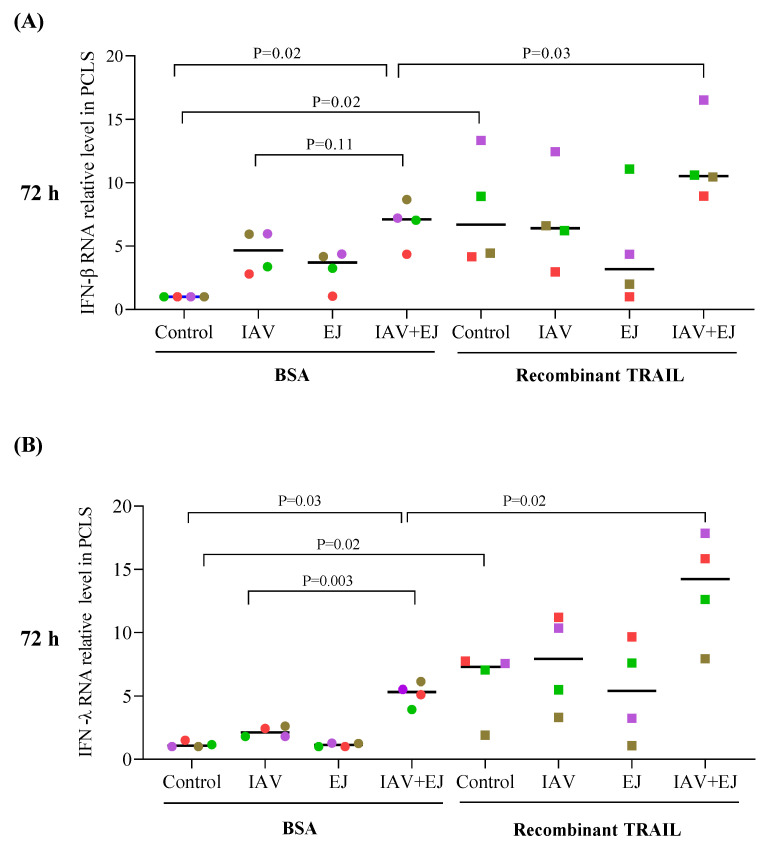
Recombinant TRAIL increased IFNs in human precision-cut lung slices (PCLS) treated with E-juice and influenza A virus (IAV). PCLS were treated with IAV and E-juice (EJ) in the presence or absence of BSA (control) and recombinant TRAIL (10 ng/mL) for 72 h. Levels of IFN-β (**A**) and IFN-λ (**B**) in the tissue were analyzed by RT-PCR. Each data point (colored dot) represents the average of 3 replicates from each respective individual donor (*n*  =  4 donors). Horizontal lines indicate medians; Mann–Whitney test was used to analyze the data.

Interferon production serves as a critical mechanism to eliminate viruses from infected cells [28]. It was also reported that TRAIL may up-regulate IFN-β and IFN-regulated genes [33,34]. To determine if TRAIL-mediated reduction of viral load in the tissue is associated with enhanced IFN responses, we measured IFN-β and -λ mRNA levels in PCLS. Recombinant TRAIL amplified IFN-β and IFN-λ mRNA expression in PCLS treated with both E-juice and IAV (Figure 5A,B). In line with the viral load data, E-juice and IAV co-treatment in the absence of recombinant TRAIL enhanced the expression of IFN-β (~6 fold over BSA control) and IFN-λ (~4.5 fold over BSA control). Interestingly, TRAIL treatment in control PCLS also increase the levels of IFN-β and IFN-λ mRNA.

## 3. Discussion

Application of the human PCLS model in our study, for the first time, clearly demonstrated the direct effects of E-cigarettes on viral load during IAV infection. Further, we explored the role of TRAIL in viral infection in the context of EC exposure. Our data suggest that E-juice significantly increased IAV levels as well as TRAIL release. TRAIL may serve as a mechanism to regulate viral infection during lung exposures to E-cigarettes.

The distal lung compartment, the major site of emphysema and loss of lung function [8,35,36], is vulnerable to environmentally hazardous agents. Various cell culture and animal models have been used to study the effect of vaping on viral infection [8,37]. However, these models do not authentically represent the microenvironment in human distal lungs [1] or cannot reflect the complexity of human genetic and physiological responses [38]. The human lung PCLS model offers several major advantages over other models including intact tissue/organ architecture, native microenvironment, organ-specific features such as metabolic activity, spatially correct interstitial matrix, and tissue homeostasis [1,39,40,41]. Novel application of the human PCLS model demonstrated that E-cigarette exposures increased the IAV load. In our recently published study using the human distal airway epithelial culture, we did not observe increased viral load following E-cigarette exposures although we found an exaggerated pro-inflammatory response [8]. This may be explained by the lack of cell–cell interactions in the airway epithelial cell culture model. For example, alveolar epithelial cells can also be infected by IAV and their responses to vaping remain unclear. Thus, PCLS preserving all types of lung cells may serve as a more physiologically relevant model to study the vaping effects and perhaps EVALI in humans [41]. A recent human clinical trial comparing the level of live-attenuated IAV in nasal lavage fluid samples from volunteers with or without E-cigarette exposure demonstrated a trend of increased viral load (not statistically different) in E-cigarette users compared to non-smoker controls [42]. We propose that such a finding may be in part related to the location (i.e., nasal) of the samples collected, and future human studies may extend the sample collection site to the distal lung area. Nonetheless, our PCLS model data supports a previous mouse study where E-cigarette exposures were shown to increase lung IAV titers [43]. Together, our human PCLS model may provide a promising platform to study the mechanisms and new therapeutic targets.

Induction of TRAIL by IAV infection has been suggested to serve as a defense mechanism to remove virus-producing cells from the tissue [22,29,44], but whether TRAIL is involved in E-cigarette-mediated amplification of IAV infection has not been investigated. Using our PCLS model, we found that the combination of IAV and E-juice further increased TRAIL and TNF-α release. To evaluate the role of TRAIL during IAV infection with or without E-cigarette exposures, TRAIL neutralizing antibody and recombinant TRAIL protein were utilized to demonstrate their effect on viral load in the tissue and supernatants, indication of viral replication and release, respectively. The complementary data from our experiments suggest that TRAIL reduced tissue viral load or replication while increasing the release of viruses into the supernatants in the absence or particularly in the presence of E-cigarette exposures. Our data supports the protective role of TRAIL in IAV infection as reported in a mouse model [22,29,44].

How TRAIL protects against viral infection remains incompletely understood. It is generally believed that TRAIL-mediated apoptosis or cell death represents one of the major mechanisms to remove cells infected with viruses [29,45,46]. In our study, E-juice alone or IAV alone slightly or moderately increased the levels of cytotoxicity. Our data is in line with previous studies showing that electronic cigarettes have less cytotoxic effects than tobacco smoke [47,48,49]. However, combination of E-cigarettes and IAV significantly reduced cell viability, suggesting that E-juice exacerbated tissue injury following viral infection. In our current study, application of TRAIL neutralizing antibody and recombinant TRAIL protein trended to decrease and increase LDH levels, respectively. This may, in part, explain the effect of TRAIL on viral infection; however, other mechanisms may also be involved. It has been shown that type I and type III IFNs induce TRAIL expression [50,51,52,53]. However, TRAIL may serve as a feedback loop mechanism to induce IFN expression in cancer cell lines without viral infection [33]. We found that E-juice-mediated amplification of viral load and TRAIL was associated with increased expression of IFN-β and IFN-λ, suggesting a potential role of IFNs in TRAIL production. Notably, our finding of increased IFN-β and IFN-λ expression following recombinant TRAIL treatment in PCLS without IAV infection indicates that TRAIL alone may promote IFN expression. In the presence of IAV infection, TRAIL further enhanced IFN-β and IFN-λ expression in lung slices treated with both E-juice and IAV. TRAIL-mediated IFN expression may represent an additional mechanism for observed changes of viral load in the lung tissue and supernatants following TRAIL treatment in PCLS exposed to E-juice and IAV.

**Figure 6:**
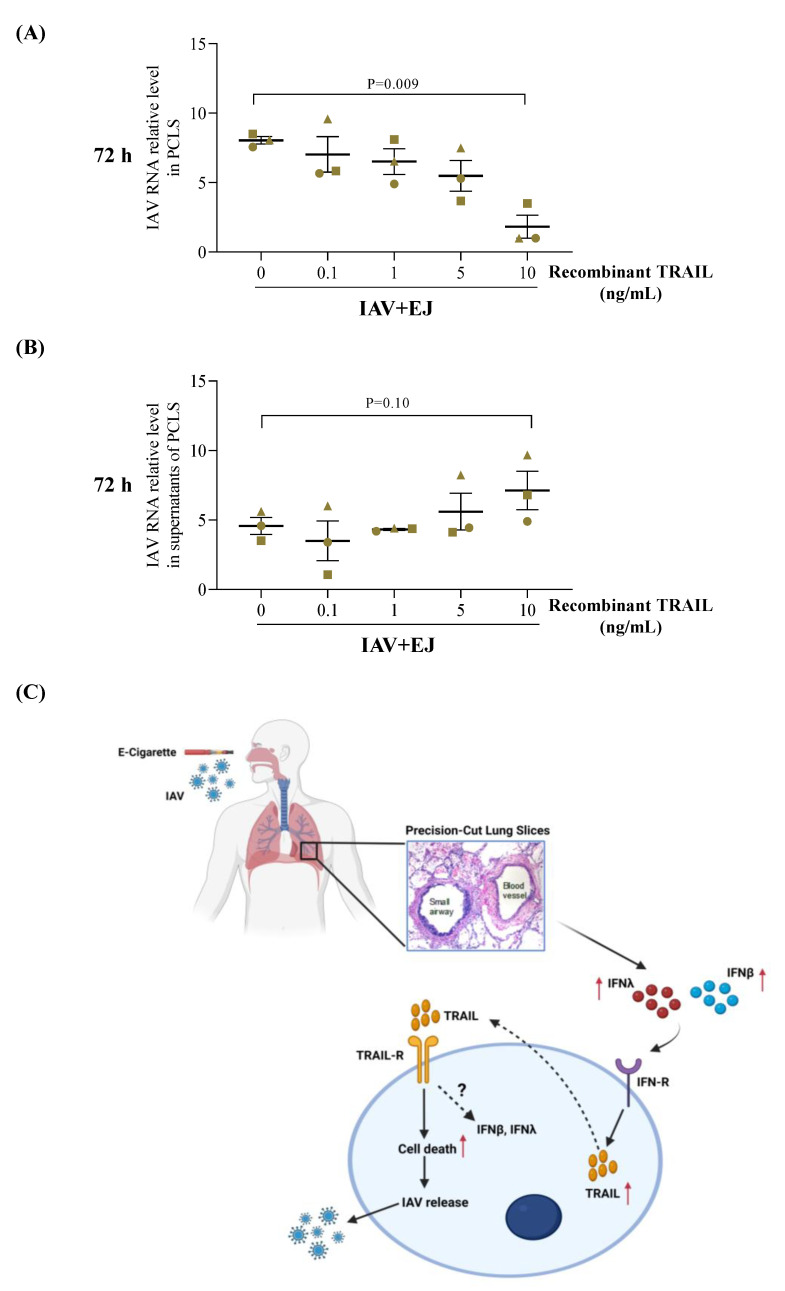
The dosing effect of recombinant TRAIL on viral infection in human precision-cut lung slices (PCLS) treated with E-juice (EJ) and influenza A virus (IAV). PCLS were treated with IAV and EJ in the presence or absence of BSA (control) and recombinant TRAIL (0.1–10 ng/mL) for 72 h. IAV load in tissue (**A**) and supernatants (**B**) was analyzed by RT-PCR. Each data point represents the individual replicate from a single donor. Horizontal lines indicate mean ± SEM. Paired *t*-test was used to analyze the data (**C**) Proposed mechanisms by which electronic cigarette (E-cig) exposures regulate IAV infection in human distal lungs. E-cig exposures amplify the expression of IFN-β and IFN-λ induced by IAV infection, which may upregulate TRAIL release. TRAIL binds to the TRAIL receptors to initiate cell apoptosis to enhance the release of IAV from the infected tissue. As a feedback loop mechanism, TRAIL may also upregulate the expression of IFN-β and IFN-λ to serve as an additional host defense mechanism.

One of the intriguing questions raised in our study is why the E-juice-mediated increase of TRAIL release in IAV-infected PCLS was associated with an increase, but not a decrease, of viral load in the tissue and supernatants. We propose that E-juice may fail to induce sufficient levels of TRAIL expression or activity to reduce viral levels. To test this possibility, we performed a pilot study where we treated PCLS with various doses of recombinant TRAIL. Only the higher dose (10 ng/mL) of recombinant TRAIL was able to reduce the viral load in the tissue (Figure 6A,B).

By using recombinant TRAIL as a standard, we performed the western blot to determine the levels of TRAIL in PCLS supernatants and found that TRAIL levels were below 10 ng/mL under all the conditions (data not shown). Our data suggests that TRAIL levels released in the media of PCLS treated with E-juice and IAV may not be sufficient to completely control IAV infection. Although we revealed the contribution of TRAIL to viral infection in EC-exposed human lung tissue, there are other potential mechanisms including the inhibitory effect of EC on host defense protein SPLUNC1 [54,55] and EC-mediated disruption of lung lipid homeostasis involved in innate immunity [56]. These additional mechanisms can be further explored in our future studies using the human PCLS model. There are several limitations to this study. First, we examined the effect of the short duration (72 h) of E-juice treatment on human PCLS, which may not reveal the long-term impact of vaping on the lung health of EC users. Second, we focused on the effect of E-cigarettes on IAV-mediated TRAIL release and subsequent IAV infection but did not explore the effect of downstream signaling of TRAIL such as apoptosis or apoptotic molecules on viral infection. Third, E-cigarettes contain proprietary components, and our study did not clarify which components were responsible for altered TRAIL expression and viral infection. There are multiple types of cells in PCLS, but we do not know exactly how each type of cell may play a role in regulating IFN and TRAIL expression and subsequently contribute to EC-mediated exacerbation of viral infection. We performed a preliminary single-cell RNA sequencing experiment using PCLS from a healthy non-smoking donor. We found that IAV significantly increased the expression of TRAIL in airway epithelial cells, alveolar type I and type II epithelial cells as well as lung macrophages. E-juice appeared to further increase TRAIL expression by alveolar epithelial cells and macrophages (Supplementary Figure S1, Supplementary Table S1) in IAV-infected PCLS although the difference was not statistically significant likely due to the use of a single subject for this analysis. Lastly, in our PCLS model, IAV and EC were not delivered through inhalation due to the technical limitation. Unlike the air–liquid interface culture model for airway epithelial cells, PCLS were cultured under the submerged condition, which may not mimic the physiological route of airway epithelial exposure to viruses and vaping products although it is relevant to the in vivo exposure of lung structural cells (e.g., endothelial cells and fibroblasts) to vaping [57,58].

## 4. Conclusions

By leveraging our access to human donor lungs, we have demonstrated that E-cigarette exposures worsened distal lung tissue viral infection, which was associated with dysregulated TRAIL and IFN expression (Figure 6C). Maintenance of appropriate host responses such as TRAIL production to viruses in E-cigarette users may be beneficial to attenuate viral infection and tissue damage.

## 5. Materials and Methods

### 5.1. Preparation of PCLS from Human Donor Lungs

The upper lobes of the right lung from healthy, non-smoking donors were obtained from the International Institute for the Advancement of Medicine (Philadelphia, PA, USA) or the Donor Alliance of Colorado (Denver, CO, USA). All the donor lungs were selected based on the non-smoking status and no history of lung disease/infection. The detailed donor demographic information is given in Table 1. Lungs were inflated with 1.5% low-melting agarose (42 °C) and sliced into consecutive 450 µm thickness sections using a Compresstome^®^ VF-300 vibratome (Precisionary Instruments, Natick, MA, USA). The slices were transferred to 24-well plates containing Dulbecco’s Modified Eagle’s Medium (DMEM, Thermo Fisher Scientific, Waltham, MA, USA) with antifungal agents and antibiotics and incubated in a humidified incubator at 37 °C supplemented with 5% CO_2_.

### 5.2. IAV Preparation

We used the pandemic influenza A/California/07/2009 virus which was initially and generously provided by Dr. Kevin Harrod from the University of Alabama at Birmingham and further propagated by Dr. Mari Numata Nakamura at National Jewish Health, Denver [59]. IAV was propagated in Madin-Darby canine kidney cells (MDCK; ATCC, Manassas, VA, USA) [8,60,61,62,63]. MDCK cells were grown in DMEM (Thermo Fisher Scientific, Waltham, MA, USA) as described previously [59]. IAV was harvested after 72 h post-infection and titered by plaque assay using MDCK cells [59].

### 5.3. IAV Infection in Human PCLS

Human PCLS were incubated with 1.5 µg/mL of TPCK-treated trypsin [control] (Thermo Fisher Scientific, Waltham, MA, USA) or IAV (3 × 10^5^ PFU/well) in 250 µL of DMEM media supplied with antibiotics for 2 h at 37 °C and 5% CO_2_. After 2 h, the virus-containing medium was removed and PCLS were washed three times with warm 1X PBS to remove the unbound virus. The dose of IAV was selected based on our previous publication [59].

### 5.4. Treatments with E-Juice, TRAIL Neutralizing Antibody, and Recombinant TRAIL in Human PCLS

E-juice with Virginia tobacco flavor from JUUL Labs (Washington D.C.), which contains nicotine at 35 mg/mL, was used in this study. 0.05% E-juice with a final nicotine concentration of 17.5 µg/mL was added to PCLS during the 2 h IAV infection and maintained after the viruses were removed from the supernatants. A previous study [64] measured nicotine in the serum samples of EC users. Serum nicotine concentrations after 5 min of EC use ranged from 5 to 45 ng/mL. Serum nicotine concentrations were determined to be about 1000 times lower than those in the airway epithelial lining fluid [64,65,66]. Therefore, our nicotine added to human PCLS at 17.5 µg/mL is considered to be within the physiological range of human EC users.

To demonstrate the role of TRAIL in viral infection, a TRAIL neutralizing antibody (50 ng/mL, Peprotech, Cranbury, NJ, USA) or an IgG antibody control (50 ng/mL, Jackson Immuno-research, West Grove, PA, USA) was added to PCLS exposed to 0.05% E-juice with or without IAV infection for up to 72 h. Similarly, recombinant human TRAIL (0.1–10 ng/mL, Peprotech, Cranbury, NJ, USA) or bovine serum albumin (BSA) was applied to PCLS exposed to E-juice with or without IAV infection. After 24, 48, and 72 h time points, supernatants and tissues were collected.

### 5.5. RT-PCR

Expression of interferon beta (IFN-β) and interferon lambda (IFN-λ) mRNA, intracellular and extracellular IAV RNA was measured by reverse transcription and quantitative real-time PCR (RT-PCR). To extract total RNA, PCLS was homogenized using the TRIzol reagent, followed by using Mini Spin Columns for RNA extraction (Epoch Life Science Inc., Missouri City, TX, USA) according to the manufacturer’s instructions. cDNA was generated through a Bio-Rad T100 thermocycler. RT-PCR was performed using a probe-based method where 18s RNA (ThermoFisher, Waltham, MA, USA) was used as a housekeeping gene. The custom-made (Integrated DNA Technologies, Coralville, IA, USA) specific primers and probes for human IFN-λ1 were forward: 5′-GGGAACCTGTGTCTGAGAACGT-3′; reverse: 5′-GAGTAGGGCTCA GCGCATAAATA-3′; probe: 5′-CTGAGTCCACCTGACACCCCACACC-3′), for IFN-β forward: 5′-GACGGAGAAGATGCAGAAGAG-3′, reverse:5′-CCACCCAGTGCTGGA GAA -3′, probe: 5′-TGCCTTTGCCATCCAAGAGAT-3′; and for IAV were forward: 5′-GAC CRATCCTGTCACCTCTGAC-3′, reverse:5′-AGGGCATTYTGGACAAAKCGTCTA-3′, probe: 5′-TGCAGTCCTCGCTCACTGGGCACG-3′. The comparative cycle of threshold (ΔΔCT) method was used with the housekeeping gene 18S rRNA as an internal control to calculate the relative gene expression levels.

### 5.6. Western Blotting

TRAIL released in PCLS supernatants was measured using western blotting. As TRAIL levels in the raw supernatants were low, supernatants were concentrated using Amicon ultra centrifugal filters (MilliporeSigma, Burlington, MA, USA). An equal volume of concentrated supernatants was separated on 15% SDS-polyacrylamide gels, transferred onto PVDF membranes, blocked with western blocking buffer, and incubated with a mouse anti-human TRAIL antibody (R&D Systems, Minneapolis, MN, USA) overnight at 4 °C. The next day, the membranes were washed in PBS with 0.1% Tween-20, incubated in HRP-conjugated IgG secondary mouse antibody (EMD Millipore, Burlington, MA, USA) followed by developing using a Fotodyne imaging system (Fotodyne, Inc., Hartland, WI, USA). Image J software (National Institutes of Health, Bethesda, MD, USA) was used to measure the intensity of TRAIL protein expression and calculate the fold change values of treatment groups versus the non-treated control groups.

### 5.7. TNF-α ELISA

TNF-α levels were measured in PCLS supernatants using a Human TNF-α DuoSet ELISA kit (R&D Systems, Minneapolis, MN, USA) according to the manufacturer’s instructions.

### 5.8. Lactate Dehydrogenase (LDH) Assay

To determine the cytotoxic effects of IAV and E-juice, LDH levels in PCLS supernatants were measured using an LDH detection kit (Roche Diagnostics, Indianapolis, IN, USA) according to the manufacturer’s instructions. Data were expressed as the fold changes of various treatment groups versus the control groups.

### 5.9. Statistical Analysis

GraphPad Prism version 8.0 software was used for all statistical analyses. For parametric tests, a Student’s *t*-test was performed for two-group comparisons. Non-parametric data were analyzed using the Mann–Whitney test for two group comparisons. *p* < 0.05 was considered statistically significant.

## Figures and Tables

**Table 1:** Subject demographics.

Subject	Gender	Age (Years)	Smoking History
1	Female	32	Non-smoker
2	Female	27	Non-smoker
3	Male	30	Non-smoker
4	Male	73	Non-smoker
5	Male	63	Non-smoker

## Data Availability

All data relevant to the study are included in the article and Supplementary Materials.

## Supplementary Materials

The following supporting information can be downloaded at: https://www.mdpi.com/article/10.3390/ijms24054295/s1.

## Abbreviations

**COPD**	Chronic obstructive pulmonary disease
**ALI**	Air liquid interface
**PCLS**	Precision cut lung slices
**ENDS**	Electronic nicotine dispensing systems
**E-Juice**	Electronic cigarette juice
**EJ**	Electronic cigarette juice
**EC**	Electronic cigarettes
**TRAIL**	Tumor necrosis factor (TNF)-related apoptosis-inducing ligand
**TNF-α**	Tumor necrosis factor-α
**IAV**	Influenza A virus
**EVALI**	E-cigarette or vaping product use-associated lung injury
**IFN-β**	Interferon beta
**IFN- λ**	Interferon lambda
**LDH**	Lactate dehydrogenase assay
**MDCK**	Madin-Darby canine kidney
