# Supplementary material for: Electronic Cigarette Exposure Increases the Severity of Influenza a Virus Infection via TRAIL Dysregulation in Human Precision-Cut Lung Slices
Source: Int J Mol Sci. 2023 Feb 21;24(5):4295. doi: 10.3390/ijms24054295 (PMC10002047; doi:10.3390/ijms24054295)
Supplement: Supplementary file 1 [file ijms-24-04295-s001.zip › ijms-2209336-supplementary.pdf]

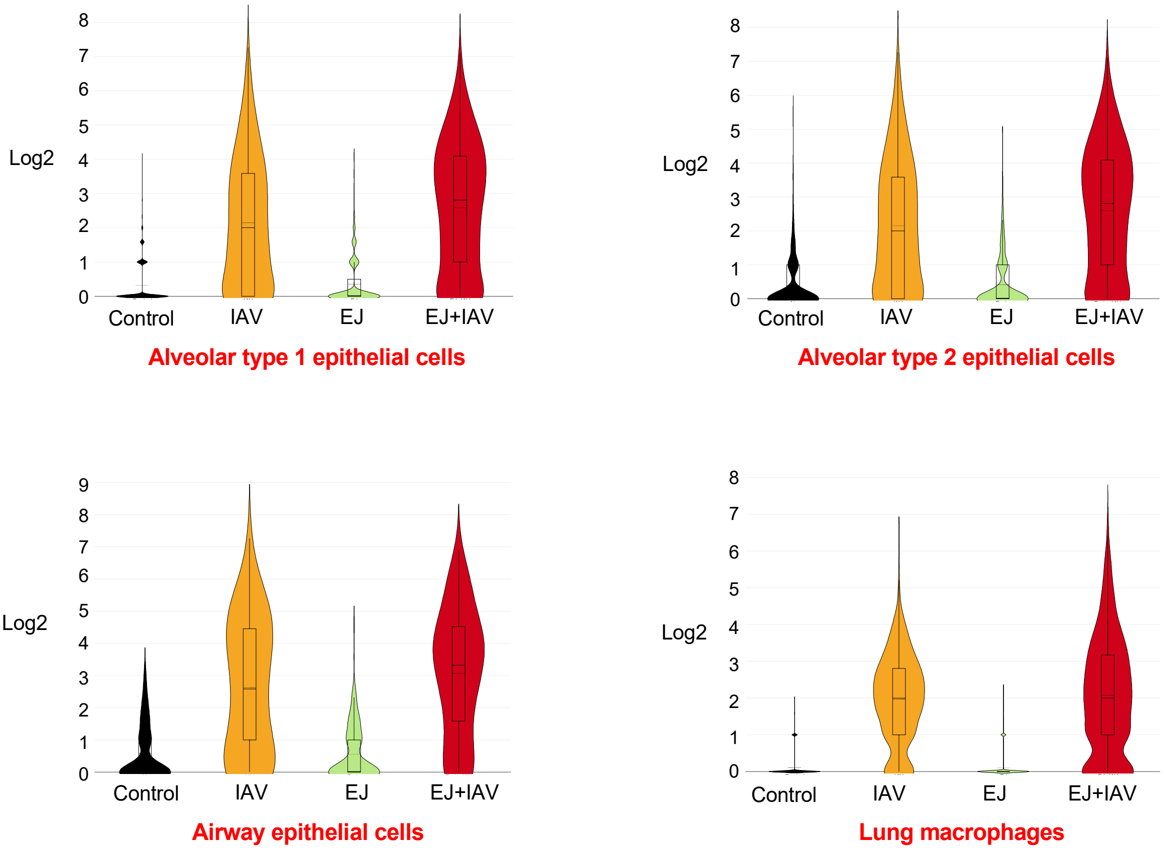

Supplementary Figure S1. Single-cell RNA sequencing data in human precision-cut lung slices demonstrate TRAIL gene expression in alveolar type 1 and type 2 cells, airway epithelial cells and lung macrophages.

Supplementary Table S1. Gene expression by different types of cells in human precision-cut lung slices after E-juice (EJ) and influenza A virus (IAV) treatment

	**EJ+IAV vs. IAV**		**EJ vs. (–)**	
Cell types	Genes	Log2 fold change	Genes	Log2 fold change
**AT1**	FGFBP1	1.93	KRT5	-2.01
	S100A2	1.76	MIR205HG	-1.90
	EGR3	1.58	FGFBP1	-1.44
	ALDH1A3	1.50	CLDN1	-1.15
	CCL3L1	1.71	SFN	-0.93
	FBXO2	1.16	KRT6A	-1.22
	AQP5	1.91	F3	-0.91
	KRT5	1.48	SERPINB4	-1.05
	MYC	1.16	KRT17	-0.79
	CLDN1	1.11	AQP3	-0.75
	SERPINB3	1.54	S100A2	-0.81
	LURAP1L	1.14	SERPINB3	-0.91
	SDC1	1.16	S100A14	-0.78
	F3	1.07		
	C1orf116	1.17		
	CCL4L2	1.66		
	GALNT5	1.08		
	LIF	1.10		
	PTGS2	0.94		
	FOXQ1	1.22		
	AQP3	0.98		
	EGFR	0.99		
	HBEGF	0.96		
	TFAP2A	0.93		
	SLC5A3	0.87		
	CYP1B1	0.83		
	ITGA2	0.85		
	MRPS6	0.82		
	PALLD	0.84		
	CLDN4	0.85		
	SPINT1	0.84		
	MMP1	1.42		
	KRT6A	0.96		
	PHLDA1	0.83		
	CCL8	1.33		
	ERRFI1	0.81		
	PDP1	0.83		
	ID1	0.88		
	GADD45A	0.79		
	CEACAM6	0.91		
	CXCL8	0.79		
	TACSTD2	0.77		
	SFN	0.75		
	SLPI	0.86		
**ATII**	FGFBP1	1.93	KRT5	-2.01
	S100A2	1.76	MIR205HG	-1.90

	EGR3	1.58	FGFBP1	-1.44
	ALDH1A3	1.50	CLDN1	-1.15
	CCL3L1	1.71	SFN	-0.93
	FBXO2	1.16	KRT6A	-1.22
	AQP5	1.91	F3	-0.91
	KRT5	1.48	SERPINB4	-1.05
	MYC	1.16	KRT17	-0.79
	CLDN1	1.11	AQP3	-0.75
	SERPINB3	1.54	S100A2	-0.81
	LURAP1L	1.14	SERPINB3	-0.91
	SDC1	1.16	S100A14	-0.78
	F3	1.07		
	C1orf116	1.17		
	CCL4L2	1.66		
	GALNT5	1.08		
	LIF	1.10		
	PTGS2	0.94		
	FOXQ1	1.22		
	AQP3	0.98		
	EGFR	0.99		
	HBEGF	0.96		
	TFAP2A	0.93		
	SLC5A3	0.87		
	CYP1B1	0.83		
	ITGA2	0.85		
	MRPS6	0.82		
	PALLD	0.84		
	CLDN4	0.85		
	SPINT1	0.84		
	MMP1	1.42		
	KRT6A	0.96		
	PHLDA1	0.83		
	CCL8	1.33		
	ERRFI1	0.81		
	PDP1	0.83		
	ID1	0.88		
	GADD45A	0.79		
	CEACAM6	0.91		
	CXCL8	0.79		
	TACSTD2	0.77		
	SFN	0.75		
	SLPI	0.86		
**Airway epithelial cells** (entire population)	S100A2	2.09	MT1X	-1.54
	FGFBP1	1.74	KRT6A	-1.46
	SERPINB3	1.79	S100A14	-1.09
	EGR3	1.72	CEACAM6	-1.22
	LGALSL	1.66	F3	-1.08
	LRRC8A	1.31	SFN	-0.88
	CLDN1	1.31	KRT5	-1.38
	GNE	1.55	MIR205HG	-1.22
	ALDH1A3	1.37		
	OVOL1	1.63		
	DUSP5	1.15		

	KRT6A	1.35		
	IGFBP3	1.37		
	AQP5	1.66		
	ST6GAL1	1.29		
	AQP3	1.03		
	KRT5	1.41		
	TRIM29	1.15		
	GADD45A	1.01		
	PHLDA1	1.22		
	KLF4	1.03		
	EPHA2	0.99		
	IL1RN	1.07		
	KRT17	0.94		
	MRPS6	1.00		
	PI3	2.13		
	PRSS23	1.19		
	PLAUR	1.01		
	RALGDS	1.04		
**Alveolar macrophages**	IL1B	0.93	S100A2	-1.43
	CCL4L2	0.88	CCL3L1	-0.82
	KRT17	0.91	KRT17	-0.93
	AKR1B1	0.78	CCL2	-0.78
	F3	0.84		
	CCL2	0.77		
	IGLC1	7.44		
	MRPS6	0.74		
	CLDN1	1.06		
	S100A2	2.23		
